# Karnofsky Performance Status and quality of life in patients with relapsed or refractory primary CNS lymphoma from a phase I/II study of tirabrutinib

**DOI:** 10.1093/noajnl/vdad109

**Published:** 2023-09-14

**Authors:** Yoshiki Arakawa, Yoshitaka Narita, Motoo Nagane, Kazuhiko Mishima, Yasuhito Terui, Hajime Yonezawa, Katsunori Asai, Noriko Fukuhara, Kazuhiko Sugiyama, Naoki Shinojima, Arata Aoi, Ryo Nishikawa

**Affiliations:** Department of Neurosurgery, Kyoto University Graduate School of Medicine, Kyoto, Japan; Department of Neurosurgery and Neuro-Oncology, National Cancer Center Hospital, Tokyo, Japan; Department of Neurosurgery, Kyorin University Faculty of Medicine, Tokyo, Japan; Department of Neuro-Oncology/Neurosurgery, Saitama Medical University International Medical Center, Saitama, Japan; Department of Hematology and Oncology, Cancer Institute Hospital, Japanese Foundation for Cancer Research, Tokyo, Japan; Department of Neurosurgery, Kagoshima University Hospital, Kagoshima, Japan; Department of Neurosurgery, Osaka International Cancer Institute, Osaka, Japan; Department of Hematology and Rheumatology, Tohoku University Graduate School of Medicine, Miyagi, Japan; Department of Clinical Oncology & Neuro-Oncology Program, Hiroshima University Hospital, Hiroshima, Japan; Department of Neurosurgery, Kumamoto University Hospital, Kumamoto, Japan; Ono Pharmaceutical Co, Ltd, Osaka, Japan; Department of Neuro-Oncology/Neurosurgery, Saitama Medical University International Medical Center, Saitama, Japan

**Keywords:** central nervous system lymphoma, Karnofsky Performance Status, quality of life, tirabrutinib, tyrosine kinase

## Abstract

**Background:**

Tirabrutinib, a second-generation inhibitor of Bruton’s tyrosine kinase, was approved in March 2020 for the treatment of relapsed or refractory primary central nervous system lymphoma (r/r PCNSL) based on phase I/II studies in Japan. We previously reported the overall response rate and safety profile. We describe Karnofsky Performance Status (KPS) and the quality of life (QoL) in patients with r/r PCNSL receiving tirabrutinib based on more than 1-year follow-up data.

**Methods:**

Patients with r/r PCNSL, age ≥20 years, and KPS ≥70 were treated with tirabrutinib once daily at a dose of 320, 480, or 480 mg under fasted conditions. QoL was assessed using questionnaires issued by the European Organization for Research and Treatment of Cancer (EORTC), namely EORTC QLQ-C30, EORTC QLQ-BN20, and EuroQol 5 dimensions 3-level (EQ-5D-3L) along with KPS.

**Results:**

Forty-four patients (mean age, 60 years [range 29–86]) were enrolled. The median follow-up period was 14.9 months (range, 1.4–27.7). The median KPS of the patients at baseline was 80.0 (range, 70–100), and this remained constant during the treatment. The global health status/QoL in the QLQ-C30 showed significant improvements from baseline through cycles 3–17 and remained relatively constant thereafter until cycle 23. Improvements were also seen in emotional functioning and constipation in the QLQ-C30 segments. Other items of QLQ-C30 and QLQ-BN20, EQ-5D visual analog scales, and EQ-5D index were maintained during the treatment.

**Conclusions:**

Tirabrutinib generally maintains KPS and QoL scores with some improvements in specific QoL items in patients with r/r PCNSL.

**Key Points**

- Karnofsky Performance Status was consistent throughout tirabrutinib treatment.
- Tirabrutinib tends to improve global health status/quality of life (QoL), emotional functioning, and constipation.
- Other dimensions of QoL were maintained without acute deterioration during treatment.

**Importance of the Study**

The incidence of primary central nervous system lymphoma (PCNSL) has increased globally and in Japan over time, with an increased incidence among the elderly. However, optimal treatment for newly diagnosed or relapsed/refractory (r/r) PCNSL has yet to be established. This exploratory analysis from an ongoing multicenter, open-label, uncontrolled phase I/II study aims to assess the effect of a BTK inhibitor on Karnofsky Performance Status (KPS) and quality of life (QoL) in r/r PCNSL. This is the first study that examines whether tirabrutinib helps Japanese patients with r/r PCNSL maintain or improve their KPS and QoL. Our findings revealed that KPS was maintained, and global health status/QoL, emotional functioning, and constipation from the QLQ-C30 were improved during tirabrutinib treatment.

Other items of QLQ-C30 and QLQ-BN20, EQ-5D VAS, and EQ-5D index were maintained during tirabrutinib treatment. Tirabrutinib may be considered a new therapeutic option to maintain or improve KPS and QoL in patients with r/r PCNSL.

Primary central nervous system lymphoma (PCNSL) is a rare extranodal, high-grade non-Hodgkin B-cell neoplasm that originates in the brain, cerebrospinal fluid, spinal cord, or eyes. While PCNSL is usually limited to the central nervous system (CNS), systemic involvement is seen in 4%–7% of patients with newly diagnosed and 10% of those with relapsed PCNSL.^1,2^

Over the last 40 years, the incidence of PCNSL has increased considerably. From 1975 to 2017, the incidence rate of PCNSL in the United States increased from 0.1/100 000 to 0.5/100 000, with an average annual percent change of 5.3%.^3^ The incidence of PCNSL has also increased in Japan in recent years and is common in older adults (50–70 years) with the highest incidence (62%) among those aged ≥60 years^4^; data from the Brain Tumor Registry of Japan (2005–2008) estimate that PCNSL accounts for 4.1% of all brain tumors,^5^ and the “Cancer Registry in Japan” reported that the annual number of patients with PCNSL was 1153 with a crude incidence rate of 0.91 per 100 000 and the median age of 71 years.^6^

Despite advances in treatment strategies, optimal treatment regimens for newly diagnosed PCNSL and relapsed/refractory (r/r) PCNSL have yet to be established. However, high-dose methotrexate (HD-MTX)-based chemotherapy is the most effective treatment and is considered a standard of care.^7^ For patients with previously untreated PCNSL, Japanese treatment guidelines recommend HD-MTX-based chemotherapy followed by whole-brain radiotherapy (WBRT).^4^ Combination therapy with rituximab, methotrexate, procarbazine, and vincristine (R-MPV) has been developed as an alternative to HD-MTX, and this treatment regimen is now becoming the standard of care for newly diagnosed PCNSL.^8^

In addition to Karnofsky Performance Status (KPS), which has been identified by Radiation Therapy Oncology Group as an independent prognostic factor^9^ and employed as a prognostic factor in clinical trials, both cognitive functioning and health-related quality of life (HRQoL) have also been identified as important outcome measures.^10^ According to guidelines proposed by the International Primary CNS Lymphoma Collaborative Group, research in PCNSL patients should emphasize cognition and HRQoL.^11^

Although survival has improved in recent decades, cognitive impairment due to neurotoxicity leading to a decline in QoL remains a risk with current treatment.^12^ Methotrexate-based chemotherapy, WBRT, or both can be neurotoxic and cause cognitive decline and leukoencephalopathy.^13,14^ Furthermore, the delayed treatment-related neurotoxicity adversely affects cognitive function and QoL, and has been reported to be more frequent in elderly patients.^15^ Hence, new treatment options that do not impair KPS and QoL are required.

Tirabrutinib is a second-generation oral Bruton’s tyrosine kinase inhibitor. Based on the findings of a phase I/II study, tirabrutinib was approved in Japan in March 2020 for the treatment of r/r PCNSL. In patients with r/r PCNSL, tirabrutinib monotherapy resulted in an overall response rate of 64% and a manageable safety profile.^16^ One-year follow-up demonstrated sustained tirabrutinib efficacy in patients with r/r PCNSL.^17^ We assessed changes in KPS and QoL during tirabrutinib treatment to confirm whether tirabrutinib also contributes to the maintenance/improvement of KPS and QoL in Japanese patients with r/r PCNSL.

## Methods

### Study Design

This was an exploratory analysis of an ongoing multicenter, open-label, uncontrolled phase I/II study conducted across 10 sites in Japan from October 2017. The data cutoff for the present interim analysis was February 2020. The study was registered at Clinicaltrials.jp under the identifier JapicCTI-173646 (https://database.japic.or.jp).

The tirabrutinib treatment protocol, inclusion and exclusion criteria, dose monitoring, and dose modification procedures are described in detail in a previous publication.^16^ As shown in Supplementary Figure 1, a total of 44 Japanese patients with r/r PCNSL, ≥20 years of age, and KPS scores of ≥70, were included in the study. Tirabrutinib was administered orally once daily as 320 mg (20 patients), 480 mg (7 patients), or 480 mg under fasted conditions (480 mg fasted, 17 patients). Tirabrutinib was administered on a 28-day cycle, and treatment was continued until disease progression or clinically unacceptable toxicity.

The study was approved by an Ethics Committee, and all patients provided written informed consent. The study was conducted in accordance with the principles of the Declaration of Helsinki and its subsequent amendments and in accordance with Good Clinical Practice guidelines.

### Questionnaires and Data Collection

KPS was assessed at the physician’s discretion during the screening period, days 1, 8, 15, and 28 of cycle 1, cycle 3, and day 1 of each cycle from that point onward until treatment discontinuation. QoL measures were evaluated using the European Organization for Research and Treatment of Cancer Quality of Life Questionnaire (EORTC QLQ-C30 [QLQ-C30], EORTC QLQ-BN20 [QLQ-BN20, brain tumor-specific]), and Euro Quality of Life Five Dimensions—3 Levels (EQ-5D-3L) questionnaires during the screening period, day 28 of cycle 1, day 1 of cycle 3, day 1 of every 2 cycles after cycle 3, and day 1 of every 4 cycles after cycle 25. The KPS and QoL evaluation times and data collection rates are shown in Supplementary Tables 1 and 2.

### Statistical Analysis

The analysis population consisted of 44 patients from the full analysis set. At each time point, the change from baseline in the QLQ-C30 functioning scales, QLQ-C30 or QLQ-BN20 symptom scales, EQ-5D visual analog scales (EQ-5D VAS), and EQ-5D index score was calculated as the mean ± standard deviation. The mixed model for repeated measures was used to evaluate the systematic transition of scale change, in which time points (cycles) and participants were used as a repeated fixed factor and a random factor, respectively. Compound symmetry, which does not consider time order, was assumed for correlation structure of changes among time points. The scale changes from baseline over time were evaluated with adjusted *P*-value using Dunnett’s multiple comparison method and the statistical significance level was set to be .05. All statistical analyses were performed with SAS version 9.4 (SAS Institute Japan Ltd).

In this study, we used the previously established minimally important difference (MID) criteria for QLQ-C30 in glioma patients to determine improvement or deterioration in patients with PCNSL.^18^Supplementary Table 3 displays the MID values. The MID criteria for glioma were used because similar criteria have not been established for PCNSL, and gliomas and PCNSL induce similar symptoms, including nausea and vomiting, confusion, vision problems, seizures, and weakness in the arms, face, or legs. The previously established MID was also used for the EQ-5D VAS and EQ-5D index.^19^ A change of 7 points from baseline was considered an improvement or deterioration for the EQ-5D VAS. A change of 0.08 points from baseline was considered an improvement or deterioration for the EQ-5D index.

## Results

### Patient Demographics and Baseline Characteristics

This exploratory analysis included 44 patients with r/r PCNSL. Baseline characteristics of patients are shown in Table 1. The median age was 60 years (range: 29–86) at baseline. The median KPS score at baseline was 80 (range: 70–100). The overall median follow-up period was 14.9 months (range: 1.4–27.7). The median follow-up periods were 19.1, 23.5, and 12.0 months for the 320, 480, and 480 mg fasted groups, respectively.

**Table 1.** Patient Demographics and Baseline Characteristics

Characteristics	All (*N* = 44)	320 mg QD (*N* = 20)	480 mg QD (*N* = 7)	480 mg fasted QD (*N* = 17)
Sex, male, *n* (%)	24 (54.5)	14 (70.0)	4 (57.1)	6 (35.3)
Age, median (range), years	60.0 (29–86)	59.5 (41–86)	54.0 (42–75)	65.0 (29–85)
KPS, *n* (%)				
<70	0	0	0	0
70	20 (45.5)	8 (40.0)	3 (42.9)	9 (52.9)
80	4 (9.1)	2 (10.0)	0 (0.0)	2 (11.8)
90	11 (25.0)	6 (30.0)	2 (28.6)	3 (17.6)
100	9 (20.5)	4 (20.0)	2 (28.6)	3 (17.6)
Median (range)	80.0 (70–100)	85.0 (70–100)	90.0 (70–100)	70.0 (70–100)
Number of previous lines of treatment, *n* (%)				
1	18 (40.9)	9 (45.0)	2 (28.6)	7 (41.2)
2–3	16 (36.4)	5 (25.0)	3 (42.9)	8 (47.1)
≥4	10 (22.7)	6 (30.0)	2 (28.6)	2 (11.8)
Median (range)	2.0 (1–14)	2.0 (1–6)	2.0 (1–14)	2.0 (1–5)
Prior therapy, *n* (%)				
Methotrexate	44 (100.0)	20 (100.0)	7 (100.0)	17 (100.0)
Rituximab	26 (59.1)	13 (65.0)	3 (42.9)	10 (58.8)
Whole-brain radiotherapy	29 (65.9)	14 (70.0)	5 (71.4)	10 (58.8)
HCT-ASCT	7 (15.9)	2 (10.0)	1 (14.3)	4 (37.8)
Disease status, *n* (%)				
Relapse^a^	33 (75.0)	17 (85.0)	2 (28.6)	14 (82.4)
Refractory^b^	9 (20.5)	3 (15.0)	3 (42.9)	3 (17.6)
Unknown	2 (4.5)	0	2 (28.6)	0
Type of CNS involvement, *n* (%)				
CSF				
Positive	9 (20.5)	1 (5.0)	1 (14.3)	7 (41.2)
Negative	35 (79.5)	19 (95.0)	6 (85.7)	10 (58.8)
IOL				
Positive	3 (6.8)	2 (10.0)	0	1 (5.9)
Minor RPE abnormality	6 (13.6)	5 (25.0)	0	1 (5.9)
Negative	35 (79.5)	13 (65.0)	7 (100.0)	15 (88.2)
GCB subtype, *n* (%)				
GCB	13 (29.5)	7 (35.0)	1 (14.3)	5 (29.4)
Non-GCB	31 (70.5)	13 (65.0)	6 (85.7)	12 (70.6)
Oncogenic mutation, *n* (%)				
* CARD11*	17 (38.6)	3 (15.0)	2 (28.6)	12 (70.6)
* MYD88*	32 (72.7)	15 (75.0)	6 (85.7)	11 (64.7)
* CD79B*	18 (40.9)	10 (50.0)	0	8 (47.1)
Sum of the products of the greatest diameters at the target lesion, mm^2^, *n* (%)				
<400	23 (52.3)	13 (65.0)	3 (42.9)	7 (41.2)
≥400	21 (47.7)	7 (35.0)	4 (57.1)	10 (58.8)
Median (range), mm^2^	385.84 (56.3–4020.5)	233.66 (56.3–2031.4)	514.99 (77.1–4020.5)	618.58 (86.5–2047.7)
Median duration of treatment (range), months	2.7 (0.8-27.6)	2.3 (0.9-27.6)	11.1 (0.8-24.7)	7.4 (0.9-14.9)
Ongoing treatment, *n* (%)	11 (25.0)	4 (20.0)	1 (14.3)	6 (35.3)
Treatment discontinued, *n* (%)	33 (75.0)	16 (80.0)	6 (85.7)	11 (64.7)
Reasons for discontinuation, *n* (%)				
Disease progression	26 (59.1)	12 (60.0)	4 (57.1)	10 (58.8)
Adverse event	3 (6.8)	1 (5.0)	2 (28.6)	0
Other^c^	4 (9.1)	3 (15.0)	0	1 (5.9)
Median follow-up (range), months	14.9 (1.4–27.7)	19.1 (4.8–27.7)	23.5 (1.4–24.8)	12.0 (2.9–15.3)

Abbreviations: CNS, central nervous system; CR, complete response; CRu, unconfirmed complete response; CSF, cerebrospinal fluid; GCB, germinal center-B-cell; HCT-ASCT, high-dose chemotherapy followed by autologous stem cell transplantation; IOL, intraocular lymphoma; KPS, Karnofsky Performance Scale; PCNSL, primary central nervous system lymphoma; PR, partial response; RPE, retinal pigment epithelium; QD, once daily.

^a^A disease that responded to the last therapy (CR, CRu, or PR) but progressed afterward was defined as relapsed PCNSL.

^b^A disease that did not respond to the last therapy (stable or progressive disease) was defined as refractory PCNSL.

^c^The investigator or sub-investigator considered it inappropriate to continue the study.

### KPS and QoL Outcomes

#### KPS.

**Figure 1.**
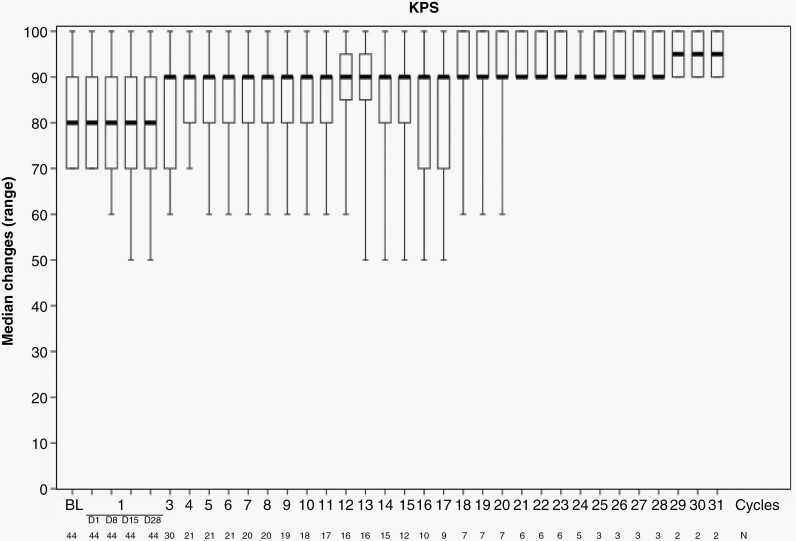
Median KPS score changes over time.

Figure 1 shows the changes in KPS during tirabrutinib treatment. The median KPS score was 80.0 (range: 70–100) at baseline and was maintained over time.

#### QLQ-C30.

**Figure 2.**
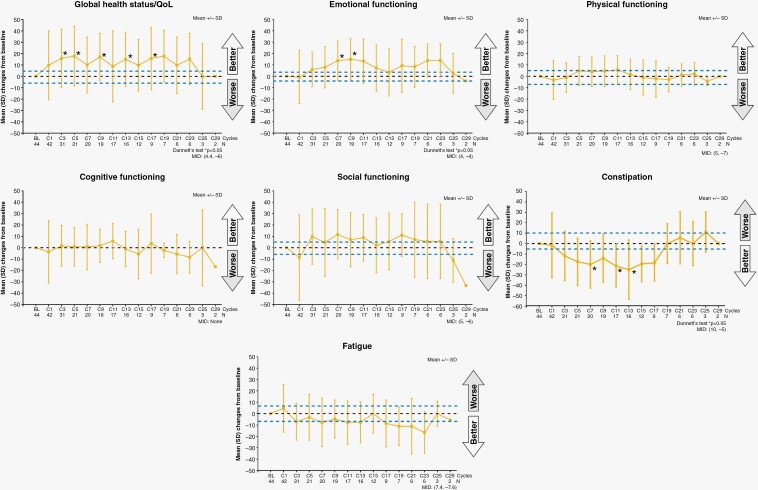
Mean changes in 7 items from the QLQ-C30 over time.

Figure 2 shows changes in 7 items from the QLQ-C30 that were of particular interest in patients with PCNSL (global health status/QoL, emotional functioning, physical functioning, cognitive functioning, social functioning, constipation, and fatigue). From cycles 3 to 17, the global health status/QoL improved significantly (*P* < .05), and the values remained higher than the baseline value until cycle 23. However, the values subsequently returned to baseline levels. From cycles 1 to 23, the values exceeded the MID.

Emotional functioning improved significantly from cycles 7 to 9 (*P* < .05) and tended to improve until cycle 23. From cycles 3 to 23, the values exceeded the MID. The constipation scale improved significantly from cycles 7 to 13 and returned to baseline levels at cycle 19. The constipation scale exceeded the MID from cycles 3 to 17. No significant differences from baseline in physical functioning, cognitive functioning, social functioning, and fatigue were observed. There were no significant differences from the baseline in any other QLQ-C30 items (Supplementary Figure 2).

#### QLQ-BN20.

**Figure 3.**
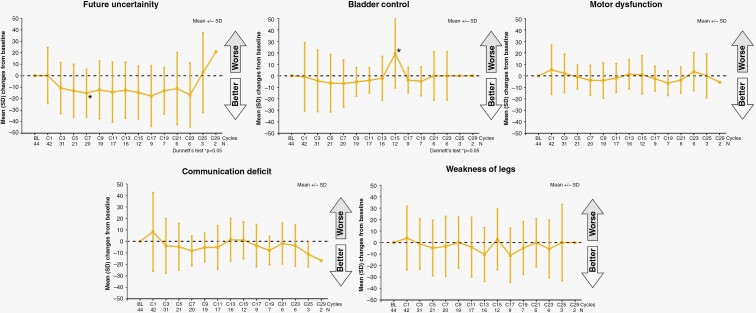
Mean changes of five items from the QLQ-BN20 over time.

Figure 3 shows the change from baseline in 5 items extracted from the QLQ-BN20 that were of particular interest in patients with PCNSL (future uncertainty, bladder control, motor dysfunction, communication deficit, and leg weakness). The future uncertainty function improved significantly only at cycle 7 and remained lower than the baseline values until cycle 23. Bladder control function deteriorated significantly at cycle 15 but did not at any other cycle. No significant differences from baseline in motor dysfunction, communication deficit, and weakness of legs were observed. Further, there were no significant differences from the baseline in the other 6 items of the QLQ-BN20 Supplementary Figure 3).

#### EQ-5D-3L.

**Figure 4.**
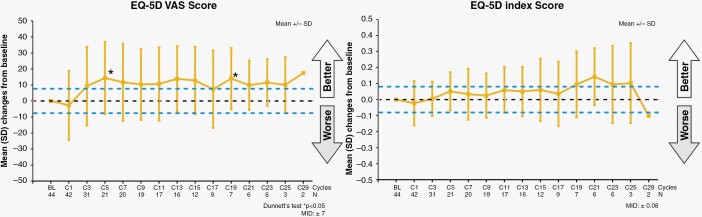
Mean changes in EQ-5D VAS and EQ-5D index over time.

EQ-5D VAS tended to improve over time and was significantly higher than baseline at cycles 5 and 19 (Figure 4). The EQ-5D VAS score exceeded the MID from cycle 3 to the final measurement. No significant differences from the baseline were observed in the EQ-5D index at any time points (Figure 4). Changes for each item of the EQ-5D-3L are shown in Supplementary Figure 4.

#### Global health status/QoL through cycle 3 by tumor response status assessed at cycle 3.

At cycle 3, tumor response was complete response (CR) or unconfirmed complete response (CRu) in 6 patients (19.4%), partial response in 11 patients (35.4%), stable disease in 5 (16.1%), and progression disease in 8 (25.8%). One patient (3.2%) was classified as “not available” due to missing the assessment. While the mean scores in global health status/QoL score increased in all tumor response groups except “not available” at cycle 1, patients with CR or CRu showed the most sustained and marked improvement through cycle 3 (Supplementary Figure 5).

#### Changes in QLQ-C30 and QLQ-BN20 in individual cases.

The chronological changes of QLQ-C30 and QLQ-BN20 in individual patients are shown as spider plot in Supplementary Figure 6. The upper panel in Supplementary Figure 6 shows the changes for ongoing treatment at data cutoff (*n* = 11) and lower panel shows the changes for discontinued patients at data cutoff (*n* = 33). Worsening was observed only in limited cases, even in those who discontinued the tirabrutinib treatment due to disease progression by cycle 3.

## Discussion

In this longitudinal study, KPS and most domains of QoL including cognitive functioning were maintained throughout treatment with tirabrutinib (median follow-up period of 14.9 months). Global health status/QoL, emotional functioning, and constipation from the QLQ-C30 improved significantly beyond the MID during tirabrutinib therapy. These data suggest that tirabrutinib could induce clinically meaningful effects on these dimensions of QoL in patients with PCNSL.

A significant improvement beyond the MID in the global health status/QoL of QLQ-C30 was observed from cycle 3 (12 weeks after the start of tirabrutinib administration). It is most likely that the improvement is correlated with the tirabrutinib efficacy, such as shrinkage of tumor and improvement of cerebral edema because 6 of 31 (19.4%) patients showed CR or CRu by physician assessment at cycle 3 and this tumor response group had the sustained and greatest improvement through cycle 3 among all tumor response groups (Supplementary Figure 5). The improvements were maintained during tirabrutinib treatment. Improvements in emotional functioning and constipation of QLQ-C30 scores are also likely associated with the shrinkage of tumors and improvement of cerebral edema induced by tirabrutinib treatment.

There was no significant difference from baseline in the other items of QLQ-C30 and QLQ-BN20 except transient changes in future uncertainty and bladder control (as measured by the QLQ-BN20). These findings imply that these dimensions were maintained during tirabrutinib treatment.

The incidence of adverse events (AEs) and treatment-related adverse events (TRAEs) was 86.4% and 81.8%, respectively, with skin disorders and hematologic disorders being the most common TRAEs. Three out of 44 patients discontinued tirabrutinib due to TRAEs. Analysis of patients with and without TRAEs revealed a shorter median treatment duration in the group without TRAEs (1.7 months vs 7.4 months with TRAEs), primarily due to early termination from disease progression. It should be noted that the shorter treatment duration group may not have had sufficient time to encounter TRAEs. Therefore, we have concluded that a direct comparison between these groups is not appropriate. Tirabrutinib has demonstrated favorable tolerability and a manageable safety profile previously.^16^ Our findings also indicate that tirabrutinib may provide benefits to patients irrespective of the presence or absence of TRAEs. This conclusion is supported by the observed maintenance or potential improvement in KPS and QoL over time, despite most patients experiencing TRAEs.

Although induction chemotherapy initially improved cognition and HRQoL, the addition of WBRT had a negative impact on both.^20^ Moreover, previous research has suggested that HD-MTX and WBRT combination therapy synergistically induces neurotoxicity.^14^ Based on our current findings, tirabrutinib therapy could be a clinically meaningful treatment option since cognitive functioning was maintained during tirabrutinib treatment in the overall population (Figure 2) as well as in a subgroup of patients (65.9%) previously receiving both methotrexate and radiation therapy (data not shown). However, the long-term effect of tirabrutinib on cognitive functioning requires further assessment.

In this study, the EQ-5D VAS score showed significant differences at 2 time points, but not at the other. However, most of the EQ-5D VAS scores were higher than the MID. Most of the EQ-5D index values, on the contrary, were closer to the baseline. Even though the EQ-5D index did not change, these findings suggest that patients’ perceptions of their current general health status may have improved to some extent.

According to Okita et al., a decline in QoL is primarily associated with lower KPS scores, leukoencephalopathy, and older age (≥65 years).^21^ It was also suggested that older patients with a history of recurrence after chemoradiotherapy are at the highest risk of QoL and KPS declines due to leukoencephalopathy development.^21^ Based on our data, it is reasonable to suggest that the maintained KPS reinforces the findings that QoL was maintained or improved during tirabrutinib treatment in the study population, including vulnerable patients such as the elderly and those with relapsed disease who had previously received both methotrexate and radiation therapy.

Our findings support the potential utility of tirabrutinib as a therapeutic option for patients with r/r PCNSL who are at risk of experiencing KPS and/or QoL decline. Further studies are needed to comprehensively evaluate the efficacy, safety, and tolerability of tirabrutinib monotherapy in improving KPS and QoL, considering a comparison with other treatments, the natural history of the disease, and the diverse ethnic backgrounds of patients with r/r PCNSL. Additionally, it is essential to investigate the long-term benefits and risks associated with tirabrutinib therapy. Herrlinger et al. demonstrated that adding WBRT therapy as consolidation therapy after chemotherapy during the initial PCNSL treatment reduced QoL and concluded that new concepts for consolidation therapy to maintain QoL after HD-MTX-based chemotherapy should be investigated.^22^ Tirabrutinib treatment, which did not worsen KPS or QoL, may fit into these new concepts. However, more research is required to determine whether tirabrutinib is a viable consolidation therapy as there is currently no evidence.

### Limitations

This was an exploratory analysis because the statistical analysis was not planned when the protocol was fixed. Statistical analysis was performed using Dunnett’s test as an additional analysis. This report is a single-arm study with a small number of cases; thus, the effects of tirabrutinib on KPS and QoL cannot be compared with those of other treatments. As the QLQ-C30 MID criteria for PCNSL have not been reported, we referred to the MID for glioma. Follow-up data on KPS and QoL after tirabrutinib discontinuation were not obtained, preventing an examination of changes compared to the continuation group. The follow-up in the present study was relatively short as a median of 14.9 months. These results need to be carefully interpreted considering the decreased number of participants whose data were assessed over time.

## Conclusions

Treatment with tirabrutinib maintains KPS and tends to improve global health status/QoL, emotional functioning, and constipation from the QLQ-C30 scores. Tirabrutinib may be considered a new therapeutic option to improve or maintain KPS and QoL in patients with r/r PCNSL.

## Supplementary Material

vdad109_suppl_Supplementary_Material
