# Supplementary material for: Karnofsky Performance Status and quality of life in patients with relapsed or refractory primary CNS lymphoma from a phase I/II study of tirabrutinib
Source: Neurooncol Adv. 2023 Sep 14;5(1):vdad109. doi: 10.1093/noajnl/vdad109 (PMC10517093; doi:10.1093/noajnl/vdad109)
Supplement: vdad109_suppl_Supplementary_Material [file vdad109_suppl_supplementary_material.zip › vdad109_suppl_Supplementary_Figures_S1-S6_Tables_S1-S3.docx]

**Supplementary Tables**

Table S1. Schedule of Questionnaire collection

Table S2. Questionnaire collection rates for each administration cycle

Table S3. MID Criteria of QLQ-C30

**Supplementary Figures**

Figure S1: Study schema

Figure S2: Mean changes in eight items from the QLQ-C30.

Figure S3: Mean changes in six items from the QLQ-BN20.

Figure S4: Mean changes in each item from EQ-5D-3L.

Figure S5: Mean change in global health status/QoL through cycle 3 by response status assessed at cycle 3.

Figure S6: Changes in A) QLQ-30 and B) QLQ-BN20 in individual cases.

**Table S1. Schedule of Questionnaire collection**

| **Visit date** | **Screening period** | **Treatment period** | | | | | | | | **Post-observation period** | **Follow-up** |
| --- | --- | --- | --- | --- | --- | --- | --- | --- | --- | --- | --- |
|  |  | DLT evaluation period | | | | | Continuous administration period | | |  |  |
|  |  | Cycle 1 | | | | | Cycle 2 | After cycle 3 | At the end of the treatment^2^  (At the time of discontinuation) | 28 days after the end of the treatment period ^2,3^ |  |
|  |  | D1 | D2 | D8 | D15 | D28 | **－** | D1**^1^** |  |  |  |
|  | －28d ~－1d | 1d | 2d | ±3d | ±3d | ±3d | － | ±7d | ±7d | ±7d |  |
| **QoL** | X |  |  |  |  | X |  | X ^4^ | X | X | X ^5^ |
| **KPS** | X | X^6^ |  | X | X | X |  | X ^7^ | X | X |  |

1. The first day of each cycle after cycle 3 was defined as [28x (number of cycles-1) + 1] days. The results performed within the acceptable time frame (±7 days) were used.
2. If the previous assessment was performed within the acceptable time frame of the end of the treatment period (discontinuation) or of 28 days after the end of the treatment period, the previous results were used. However, if more than 7 days have passed since the last assessment, the assessment was carried out. If the assessment was judged to be medically necessary, it was carried out as needed.
3. If subsequent therapy for PCNSL was started due to clinical need by 28 days after the end of the treatment period, the assessment was performed before the initiation of the subsequent therapy and the result was handled as a data for 28 days after the end of the treatment period.
4. After cycle 3, the assessment was carried out every 2 cycles (cycle 3, 5, 7.), And after cycle 25, the assessment was carried out every 4 cycles (cycle 25, 29, 33...).
5. For participants who terminated the treatment period due to safety reasons despite being judged as CR, CRu, PR, or SD, QoL assessments were performed every 8 weeks as much as possible until subsequent therapy for PCNSL was initiated or PD or recurrence was determined.
6. Performed before TIR administration.
7. After cycle 37, an assessment is carried out every 6 cycles (cycles 37, 43, 49...).

DLT, Dose-limiting toxicity; QoL, Quality of life; KPS, Karnofsky Performance Scale.

**Table S2. Questionnaire collection rates for each administration cycle, SAF (safety analysis population)**

|  | | | | |
| --- | --- | --- | --- | --- |
| **Time of assessment** | **Number of patients** | **Collecting rate (%)** | | |
|  |  | **KPS** | **EORTC^1^**  **(median)** | **EQ-5D^2^**  **(median)** |
| Screening | 44 | 100.0 | 100.0 | 100.0 |
| Cycle 1 Day 1 | 44 | 100.0 | - | - |
| Cycle 1 Day 8 | 44 | 100.0 | - | - |
| Cycle 1 Day 15 | 44 | 100.0 | - | - |
| Cycle 1 Day 28 | 44 | 100.0 | 95.5 | 95.5 |
| Cycle 3 Day 1 | 32 | 93.8 | 96.9 | 96.9 |
| Cycle 4 Day 1 | 23 | 91.3 | - | - |
| Cycle 5 Day 1 | 21 | 100.0 | 100.0 | 100.0 |
| Cycle 6 Day 1 | 21 | 100.0 | - | - |
| Cycle 7 Day 1 | 20 | 100.0 | 100.0 | 100.0 |
| Cycle 8 Day 1 | 20 | 100.0 | - | - |
| Cycle 9 Day 1 | 20 | 95.0 | 95.0 | 95.0 |
| Cycle 10 Day 1 | 18 | 100.0 | - | - |
| Cycle 11 Day 1 | 17 | 100.0 | 100.0 | 100.0 |
| Cycle 12 Day 1 | 16 | 100.0 | - | - |
| Cycle 13 Day 1 | 16 | 100.0 | 100.0 | 100.0 |
| Cycle 14 Day 1 | 15 | 100.0 | - | - |
| Cycle 15 Day 1 | 12 | 100.0 | 100.0 | 100.0 |
| Cycle 16 Day 1 | 10 | 100.0 | - | - |
| Cycle 17 Day 1 | 9 | 100.0 | 100.0 | 100.0 |
| Cycle 18 Day 1 | 7 | 100.0 | - | - |
| Cycle 19 Day 1 | 7 | 100.0 | 100.0 | 100.0 |
| Cycle 20 Day 1 | 7 | 100.0 | - | - |
| Cycle 21 Day 1 | 7 | 85.7 | 85.7 | 85.7 |
| Cycle 22 Day 1 | 6 | 100.0 | - | - |
| Cycle 23 Day 1 | 6 | 100.0 | 100.0 | 100.0 |
| Cycle 24 Day 1 | 5 | 100.0 | - | - |
| Cycle 25 Day 1 | 4 | 75.0 | 75.0 | 75.0 |
| Cycle 26 Day 1 | 3 | 100.0 | - | - |
| Cycle 27 Day 1 | 3 | 100.0 | - | - |
| Cycle 28 Day 1 | 3 | 100.0 | - | - |
| Cycle 29 Day 1 | 2 | 100.0 | 100.0 | 100.0 |
| Cycle 30 Day 1 | 2 | 100.0 | - | - |
| Cycle 31 Day 1 | 2 | 100.0 | - | - |

EORTC, European Organization for Research and Treatment of Cancer; EOT, end of treatment; EQ-5D, Euro Quality of life five dimensions. KPS, Karnofsky Performance Scale.

1. Collecting rate for EORTC was calculated as a response rate to total number of questions (50 items) in QLQ-C30 and QLQ-BN20 for each participant.
2. Collecting rate for EQ-5D was calculated as a response rate to total number of questions (5 items) for each participant.

**Table S3. MID Criteria of QLQ-C30**

| **Items** | **Improvement** | **Deterioration** |
| --- | --- | --- |
| Global health status/QoL | 4.4 | -6 |
| Physical functioning | 5 | -7 |
| Role functioning | 8 | -9 |
| Emotional functioning | 4 | -4 |
| Cognitive functioning | none | none |
| Social functioning | 5 | -6 |
| Fatigue | 7.6 | -7.4 |
| Nausea and vomiting | 6.42 | -7 |
| Pain | 7 | -6 |
| Dyspnea | 6.64 | -8 |
| Insomnia | none | none |
| Appetite loss | 9.22 | -7.5 |
| Constipation | 5 | -10 |
| Diarrhea | none | none |
| Financial difficulties | none | none |

MID, Minimal important difference; QoL, Quality of life.

**Fig. S1**. **Study schema.**

**Fig. S2**. **Mean changes in eight items from the QLQ-C30.**

BL, Baseline; QLQ, Quality of Life Questionnaire; MID, Minimal important difference; SD, Standard deviation.

**Fig. S3. Mean changes in six items from the QLQ-BN20**.

BL, Baseline; QLQ-BN, Quality of Life Questionnaire- Brain Neoplasm; SD, Standard deviation.

**Fig. S4.** **Mean changes in each item from EQ-5D.** BL, Baseline; EQ-5D, Euro Quality of life five dimensions.

**Fig. S5.** **Mean change in global health status/QoL through cycle 3 by response status assessed at cycle 3**. CR/CRu, Complete response/unconfirmed complete response; PR, Partial response; QoL, Quality of life five dimensions; SD, Stable disease.

**Fig. S6. Changes in A) QLQ-30 and B) QLQ-BN20 in individual cases**

BL, Baseline; QLQ, Quality of Life.
