# Supplementary figures and images for: Karnofsky Performance Status and quality of life in patients with relapsed or refractory primary CNS lymphoma from a phase I/II study of tirabrutinib
Source: Neurooncol Adv. 2023 Sep 14;5(1):vdad109. doi: 10.1093/noajnl/vdad109 (PMC10517093; doi:10.1093/noajnl/vdad109)

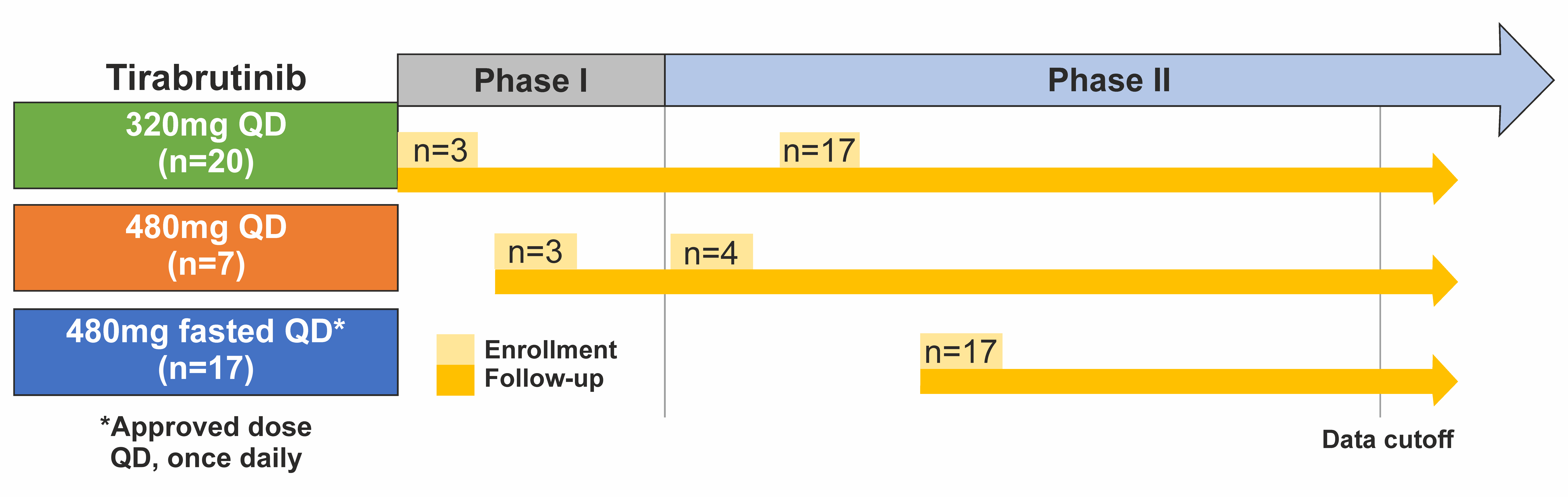

Supplement: vdad109_suppl_Supplementary_Material [file vdad109_suppl_supplementary_material.zip › Figure-S1.TIF]

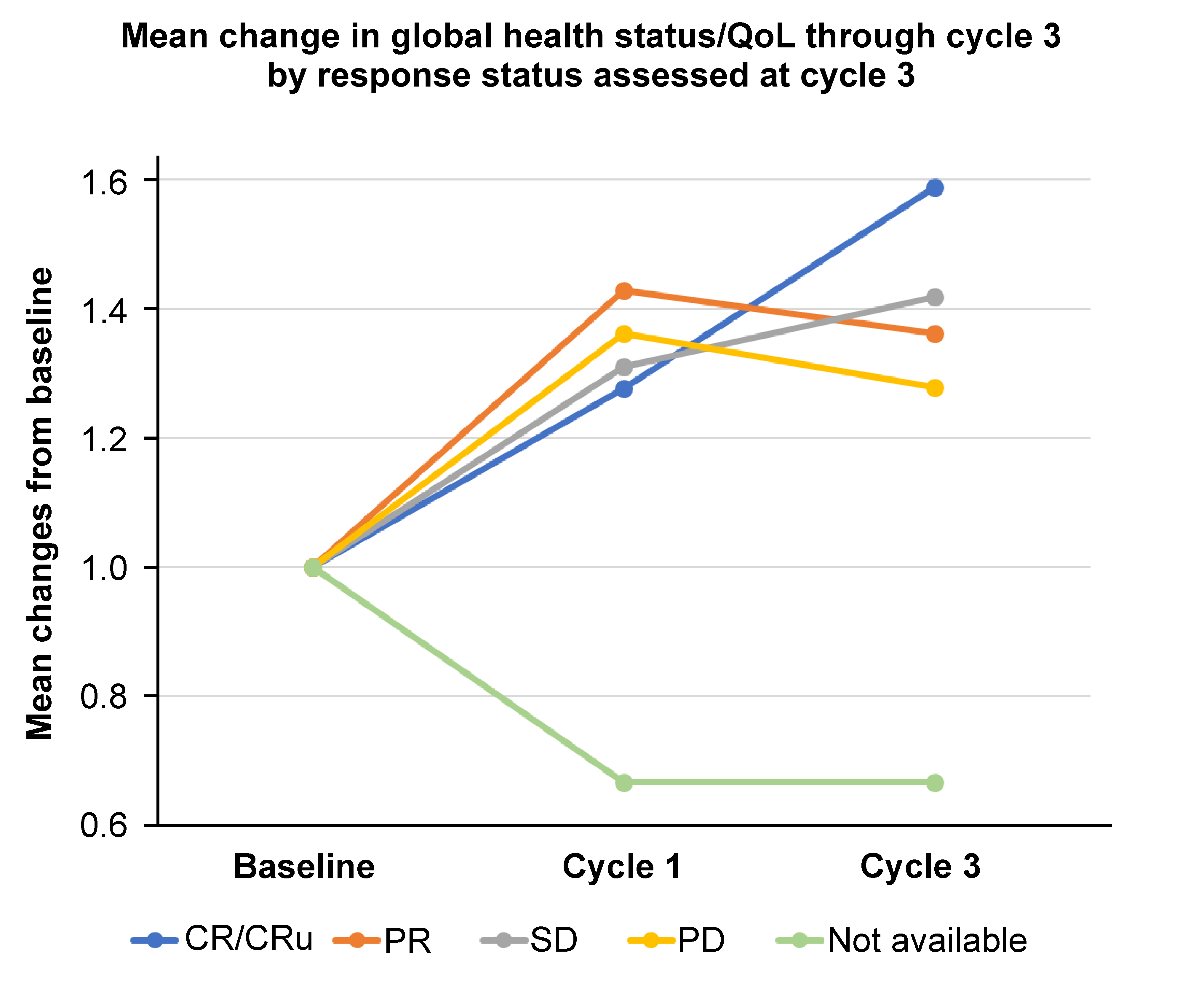

Supplement: vdad109_suppl_Supplementary_Material [file vdad109_suppl_supplementary_material.zip › Figure-S5.tif]

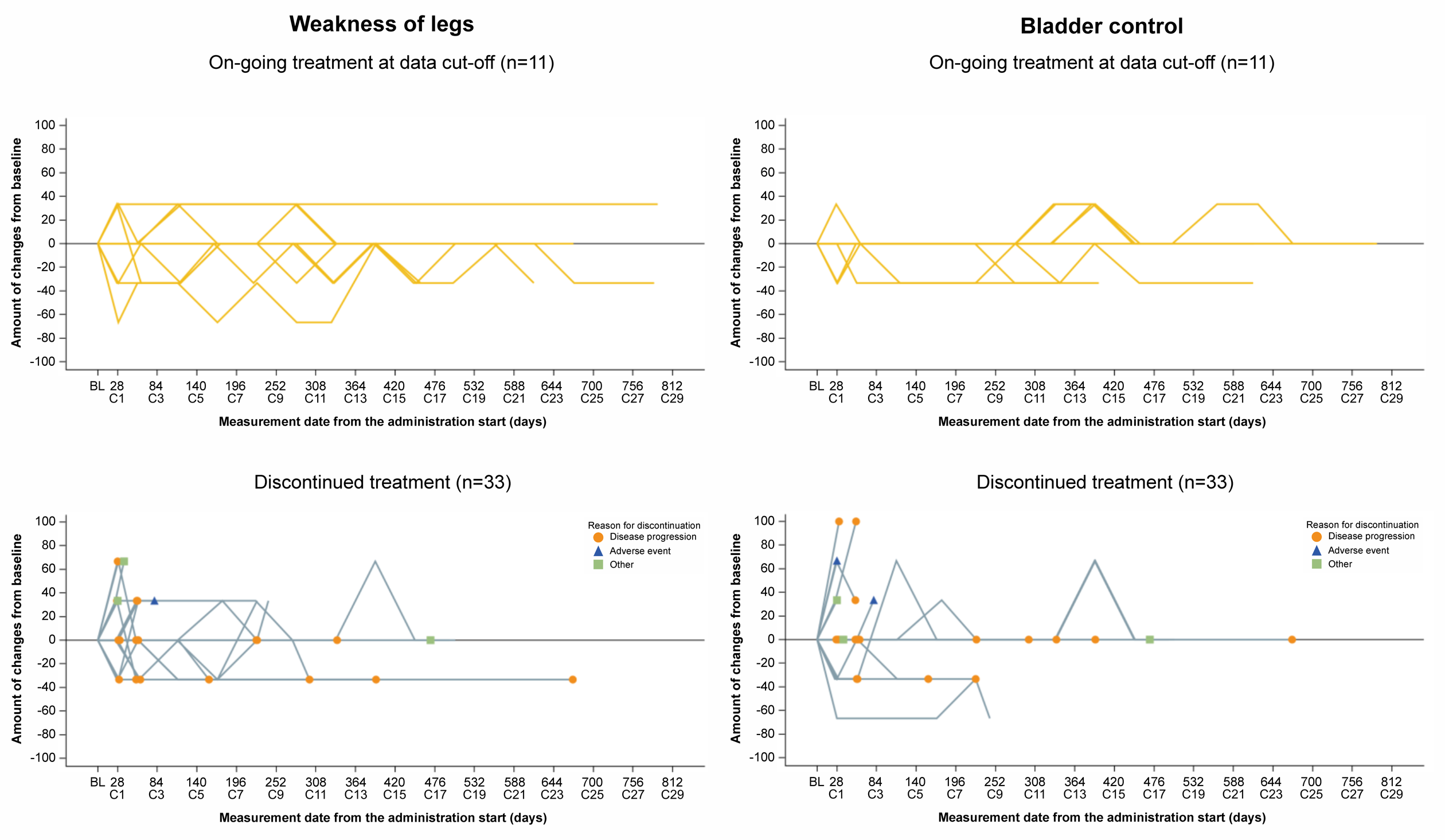

Supplement: vdad109_suppl_Supplementary_Material [file vdad109_suppl_supplementary_material.zip › Figure-S6B_9.tif]
